# *Drosophila* GATA Factor Serpent Establishes Phagocytic Ability of Embryonic Macrophages

**DOI:** 10.3389/fimmu.2018.00266

**Published:** 2018-03-08

**Authors:** Evgeny Shlyakhover, Boris Shklyar, Ketty Hakim-Mishnaevski, Flonia Levy-Adam, Estee Kurant

**Affiliations:** ^1^Department of Genetics and Developmental Biology, Faculty of Medicine, The Rappaport Family Institute for Research in the Medical Sciences, Technion – Israel Institute of Technology, Haifa, Israel; ^2^Department of Human Biology, Faculty of Natural Sciences, University of Haifa, Haifa, Israel

**Keywords:** *Drosophila*, macrophages, phagocytosis, apoptosis, SIMU, Serpent, GATA, development

## Abstract

During *Drosophila* embryogenesis, a large number of apoptotic cells are efficiently engulfed and degraded by professional phagocytes, macrophages. Phagocytic receptors Six-Microns-Under (SIMU), Draper (Drpr) and Croquemort (Crq) are specifically expressed in embryonic macrophages and required for their phagocytic function. However, how this function is established during development remains unclear. Here we demonstrate that the key regulator of *Drosophila* embryonic hemocyte differentiation, the transcription factor Serpent (Srp), plays a central role in establishing macrophage phagocytic competence. Srp, a homolog of the mammalian GATA factors, is required and sufficient for the specific expression of SIMU, Drpr and Crq receptors in embryonic macrophages. Moreover, we show that each of these receptors can significantly rescue phagocytosis defects of macrophages in *srp* mutants, including their distribution in the embryo and engulfment of apoptotic cells. This reveals that the proficiency of macrophages to remove apoptotic cells relies on the expression of SIMU, Crq and/or Drpr. However, Glial Cells Missing (GCM) acting downstream of Srp in the differentiation of hemocytes, is dispensable for their phagocytic function during embryogenesis. Taken together, our study discloses the molecular mechanism underlying the development of macrophages as skilled phagocytes of apoptotic cells.

## Introduction

During normal development of multicellular organisms superfluous cells are eliminated through apoptosis and subsequent phagocytosis by “professional” phagocytes, macrophages and immature dendritic cells, and “non-professional” tissue-resident neighboring cells (1–3). Phagocytes efficiently remove apoptotic cells with high level of specificity, which is achieved through an ability of transmembrane phagocytic receptors or secreted bridging molecules to recognize “eat me” signals exposed on the surface of apoptotic cells (4–10). Most of the phagocytic receptors are exclusively expressed in phagocytic cells, however, how their specific expression is regulated during development remains poorly understood.

*Drosophila* “professional” phagocytes macrophages (plasmatocytes) are the most abundant cells in *Drosophila* hemolymph (~95%), which similarly to mammalian macrophages are responsible for phagocytosis of apoptotic cells, microbes and tissue remodeling (11–15). They originate from the cephalic mesoderm in the embryo and remain in circulation throughout all stages of development (12, 16). The ability of macrophages to phagocytose apoptotic cells is mediated by several receptors such as Croquemort (Crq), a member of the CD36 superfamily (17, 18), Six-Microns-Under (SIMU), *Drosophila* homolog of Stabilin-2 (19–21) and Draper (Drpr), *Drosophila* homolog of MEGF10 and Jedi (2, 22–25). During embryogenesis Crq is expressed mostly in macrophages whereas SIMU and Drpr are expressed both in macrophages and in “non-professional” phagocytes glia and ectoderm (19). Our previous study demonstrated that the specific expression of SIMU and Drpr in glia is part of the developmental program responsible for glial cell differentiation (26). However, how the expression of SIMU and Drpr is regulated in macrophages remains unknown.

Serpent (Srp) is a key regulator of macrophage development during embryogenesis (27, 28). Its two isoforms, SrpC and SrpNC, are required for proper differentiation of plasmatocytes (28). *srp* mutant embryos contain lower number of macrophages, which are abnormally distributed throughout the embryo (27). Transcription factors Glial Cells Missing (GCM) and GCM2 are involved in differentiation of embryonic macrophages downstream of Srp (28). *gcm,gcm2* double mutants contain a reduced number of macrophages as well (29). However, we have shown previously that in *gcm,gcm2* mutants the expression of the phagocytic receptors SIMU, Drpr and Crq is not altered in the remaining hemocytes (26).

In the work presented here, we demonstrate that Srp is required for apoptotic cell clearance by embryonic macrophages through regulation of SIMU, Drpr and Crq expression in these cells. In addition, we show that Srp is sufficient to drive SIMU and Drpr ectopic expression. We also found that expression of each phagocytic receptor, SIMU, Drpr or Crq, alone in *srp* mutant macrophages is sufficient to partially rescue their phagocytic skills and distribution, revealing the crucial role each receptor plays in establishment of cell phagocytic ability. However, our data disclose that GCM and GCM2 are dispensable for the phagocytic clearance of apoptotic cells by embryonic macrophages.

## Materials and Methods

### Fly Strains and Constructs

The following fly strains were used in this work: *srpGal4, UAScytGFP* (I. R. Evans), *UASsrpNC and UASsrpC-FLAG/cyo* (J. Casanova, K. Campbell and M. Haenlin), *repoGal4* (B. Jones), *srp^3^/TM3* (#2485; Bloomington), *UASdrpr* (M. Freeman), *UASgcm* (#5446; Bloomington), *UASsimu* (30), *UAScrq* (ORF collection), *tubGal80^ts^* (#7019; Bloomington), *gcm-lacZ* (#5445; Bloomington), *simu-cytGFP* (19), *Df(2L)Exel7042* (#7812; Bloomington). *repoGal4::UASsrp; tubGal80^ts^* crosses were placed at 18^o^C and third instar larvae were transferred to 29°C for 14 hours.

Reporter constructs were generated by cloning different parts of a 2 kb DNA region upstream of the *simu* ORF, which recapitulates *simu* embryonic expression in all phagocytic cell populations (glia, macrophages and ectoderm) (19) into the pattB vector containing a cytoplasmic GFP coding sequence. These transgenic constructs were inserted into the attP51C site on chromosome 2R using the QC31 system (31). All strains were raised at 25°C.

### Bioinformatic Analysis

The 650 bp sequence was analyzed in Genomatix Mathinspector tool for known *Drosophila melanogaster* and vertebrate transcription factors binding sites. Only results with matrix similarity greater than 0.7 were selected. Ci value of the results was greater than 60.

### Immunohistochemistry and Live Imaging

For immunohistochemistry embryos were fixed and stained according to standard procedures. Guinea pig anti-SIMU (30) and guinea pig anti-Drpr (32) were used at a 1:5000 and 1:100 concentrations, respectively. Rabbit anti-activated caspase 3 (Dcp-1) (Cell Signaling) and mouse anti-GFP (Roche) were used at 1:100 concentration. Rabbit anti-Crq antibody (1:500) is a gift from N. Franc. Rabbit anti-Srp antibody (1:100) is a gift from J. Casanova, K. Campbell and N. Martin. Rabbit anti-Peroxidasin antibody (1:2000) is a gift from Jiwon Shim. Fluorescent secondary antibodies (Cy3/and Cy5/Jackson ImmunoResearch; Alexa Fluor 488/Molecular Probes) were used at 1:200 dilutions. For TUNEL labeling embryos were re-fixed, washed and labeled with the *In Situ* Cell Death Detection kit (Roche) according to the manufacture instructions. Images were acquired on a confocal microscope Zeiss LSM 700 or on a Zeiss Axio Observer microscope equipped with an Apotome system using the AxioVision software. 75% Glycerol solution was used as the imaging medium.

Live imaging was carried out by dechorionating embryos (stage 15), mounting them under Halocarbon oil, injecting 2–3% egg volume of LysoTracker (Molecular Probes) as described in Ref. (33). Recording started 30 min following injection.

### Statistical Analysis

For statistical analysis in each embryo number of apoptotic particles was quantified inside 10 macrophages that contain at least one apoptotic particle. 5–8 embryos of each genotype were tested (*n* = number of embryos, indicated in each figure legend). The average number of apoptotic particles per macrophage (“phagocytic index”) was calculated per embryo by dividing the total number of apoptotic particles inside labeled macrophages by the number of macrophages taken into account in this embryo. Significance was calculated by an unpaired Student’s *t*-test or by one-way ANOVA followed by Bonferroni *post hoc* test.

To count the number of REPO-positive nuclei, apotome stacks (19 µm) were acquired from the whole CNS followed by Image analysis of the designated area using IMARIS (Bitplane) software.

## Results

### *srp* Is Required for Expression of SIMU in Embryonic Macrophages

We have previously shown that during embryogenesis *simu* expression is differentially regulated in macrophages and glia; GCM directly controls *simu* transcription in glia, but not in macrophages (26). Therefore, how *simu* expression is regulated in embryonic macrophages remained unclear.

**Figure 1:**
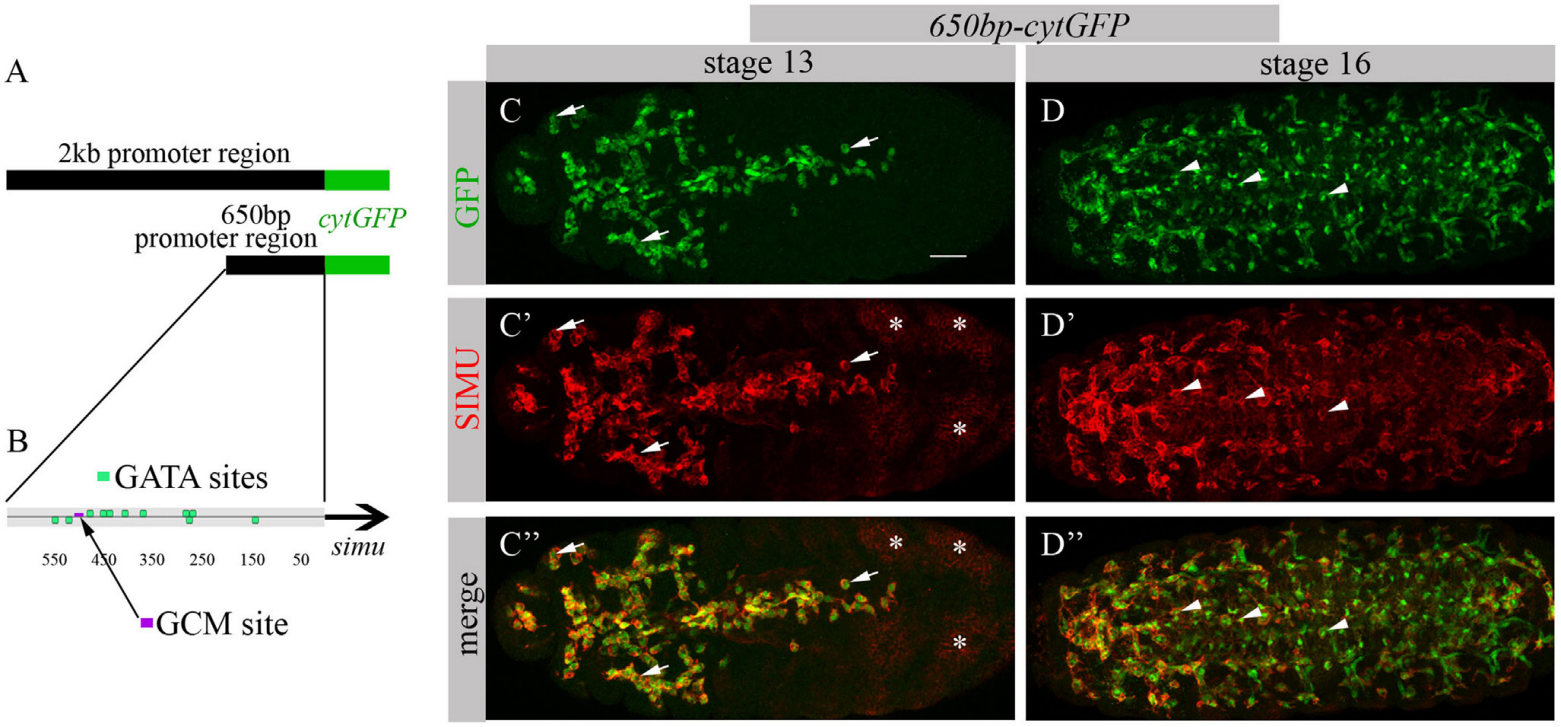
650 bp region upstream to *simu* ORF recapitulates *simu* endogenous embryonic expression and contains multiple GATA binding sites. **(A)** Schematic of 2 kb region of *simu* promoter fused to cytoplasmic GFP sequence. **(B)** Schematic map of 650 bp region of *simu* promoter fused to cytoplasmic GFP sequence with depicted putative GATA sites and one GCM binding site. **(C–D’’)** Projections from confocal stacks of the stage 13 **(C-C’’)** and stage 16 **(D–D’’)** embryos, ventral view. **(C,C’’,D,D’’)** Cytoplasmic GFP reporter and **(C’,C’’,D’,D’’)** SIMU protein as detected on membranes with anti-SIMU antibody. Bar, 20 µm. Note colocalization of GFP and SIMU in macrophages (arrows) and glia (arrowheads) but not in ectoderm (stars).

To identify factors responsible for SIMU expression in macrophages, we decided to limit our search to the smallest regulatory unit responsible for SIMU expression in these cells. For that, we reduced a 2 kb DNA region upstream of the *simu* ORF that directs cytoplasmic GFP expression in all phagocytic cell populations (glia, macrophages and ectoderm) (19) (Figure 1A) to a series of smaller overlying fragments. These fragments of the 2 kb regulatory region, fused to cytoplasmic GFP, were used for transfection in S2 cells and/or for generation of transgenic flies. A 650 bp fragment (Figure 1A) was found as the minimal region that drives GFP expression in S2 cells, as well as in macrophages and glia in the embryo, as shown by a complete overlap of anti-GFP and anti-SIMU labeling (Figures 1C–D’’). The 650 bp fragment contains one GCM binding site (Figure 1B), which explains GFP expression in glia. Smaller fragments were not able to induce any GFP expression in S2 cells. We applied the 650 bp sequence to the Genomatix software to identify binding motifs of known transcription factors.

**Figure 2:**
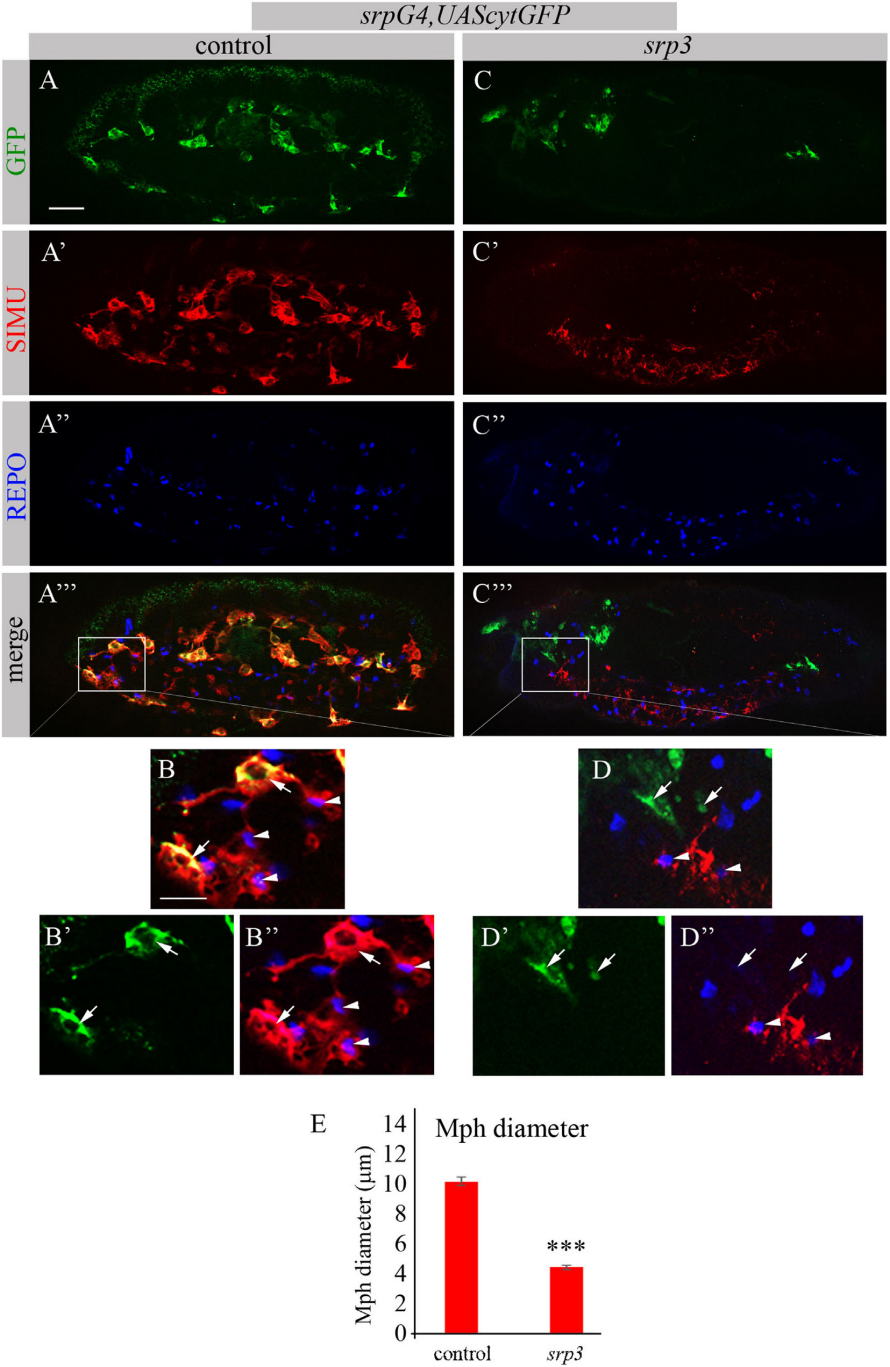
SIMU is not expressed in *srp* mutant macrophages. **(A–D’’)** Projections from confocal stacks of the stage 16 embryos, lateral view. Macrophages are labeled with *srpGal4,UAScytGFP* (green). Anti-SIMU in red. Glial nuclei are labeled with anti-REPO (blue). **(A–B’’)** Control *srpGal4,UAScytGFP* embryo. **(C–D’’)** *srpGal4,UAScytGFP*; *srp^3^* mutant embryo. **(B–B’’,D–D’’)** Close up of rectangle areas in **(A’’’,C’’’)** respectively. All GFP-positive macrophages express SIMU on their membranes in control embryo [**(B,B’’)**, arrows] but no one expresses SIMU in *srp* mutant embryo [**(D,D’’)**, arrows]. Note SIMU expression in glia (non GFP-positive cells, arrowheads). Bar, 20 µm. **(E)** Columns represent mean diameter of 10 macrophages in each embryo ± SEM. Control embryos (*n* = 5). *srp^3^* mutant embryos (*n* = 7). Asterisks indicate statistical significance versus control, as determined by Student’s *t*-test, ***p < 0.0001.

The Genomatix software identified more than 50 different sites, which have been further evaluated by the expression pattern of the corresponding transcription factors. From these potential regulators we focused on three most promising candidates: *dSTAT, pangolin* and *srp*, since they are all expressed in embryonic macrophages at stages when *simu* expression originates (Flybase data base). To examine whether these factors are required for *simu* expression, we tested SIMU expression in mutant embryos of each candidate, using the anti-SIMU antibody. *stat92E* and *pangolin* mutant embryos exhibited normal SIMU staining in embryonic macrophages (results not shown), however, *srp* mutant embryos containing a strong hypomorph mutation (*srp^3^*) (27) did not reveal detectable SIMU staining in embryonic macrophages labeled with a *srpGal4,UAScytGFP* marker (Figures 2C–D’’). *srp* mutant embryos exhibited significantly smaller macrophages as evaluated by measuring their diameter (Figure 2E), which were abnormally distributed throughout the embryo compared to control (Figure 2A) and often clustered in the anterior part of the embryo (Figure 2C). Importantly, the CNS of *srp* mutant embryos was also deformed as visualized with a specific marker for glial cells, an anti-REPO antibody (Figure S1 in Supplementary Material). However, the number of glial cells was not different from control embryos (Figure S1 in Supplementary Material) and SIMU expression was detected in relatively normal levels in glial cells (Figures 2A’–A’’’,C’–C’’’). Together, these data demonstrate that Srp is required for SIMU expression in embryonic macrophages.

### *srp* Is Required for the Phagocytic Function of Embryonic Macrophages

**Figure 3:**
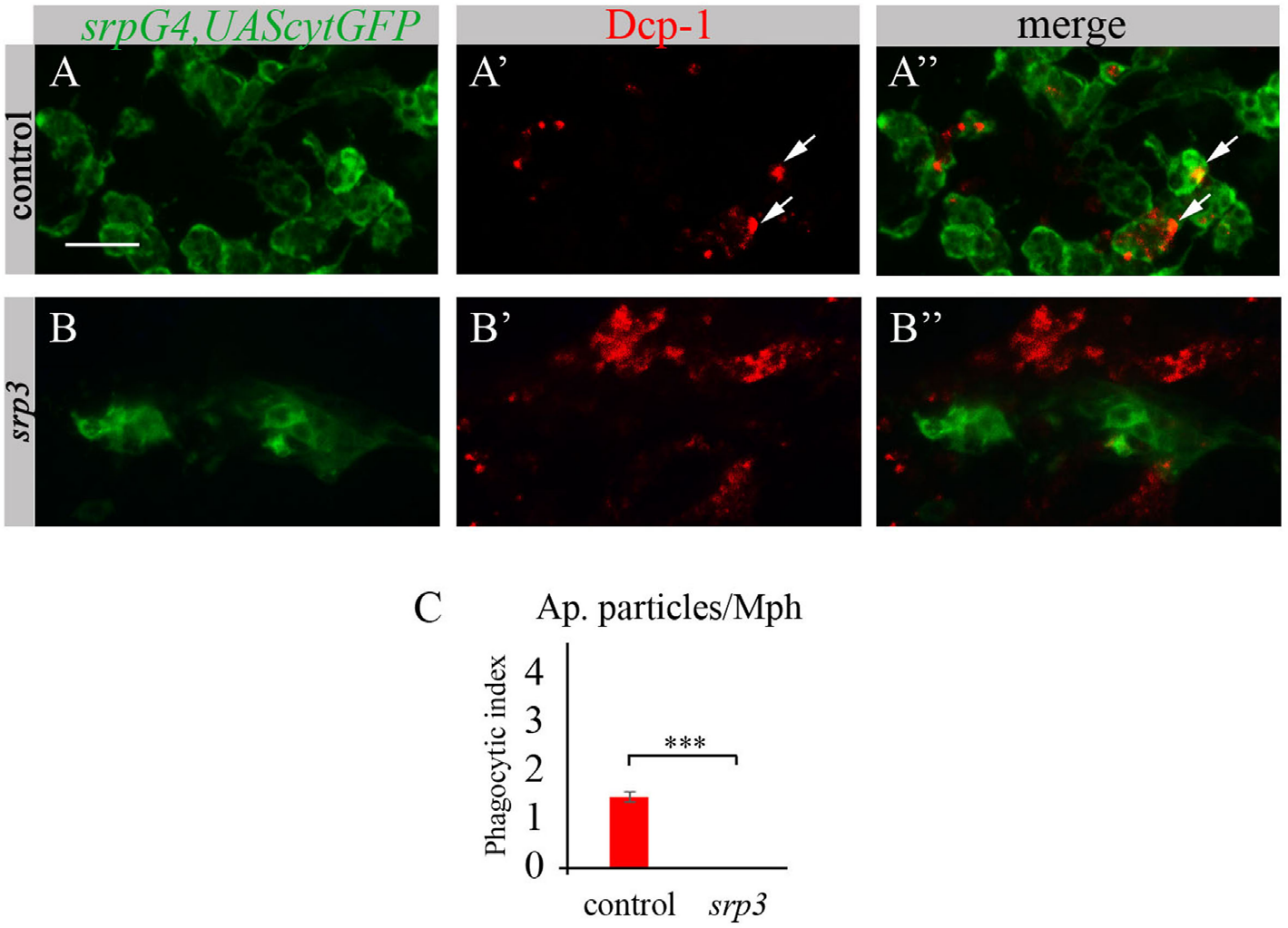
Srp is required for phagocytic ability of embryonic macrophages. **(A–B’’)** Projections from confocal stacks of the stage 16 embryos, ventral view, anterior region. Macrophages are labeled with *srpGal4,UAScytGFP* (green) and apoptotic particles are labeled with anti-Dcp-1 (red). In control *srpGal4,UAScytGFP* embryo **(A–A’’)** apoptotic particles are mostly inside GFP-positive macrophages (arrows). In *srp^3^* mutant embryo **(B–B’’)** all apoptotic particles are outside GFP-positive macrophages **(B’’)**. Bar, 20 µm. **(C)** Quantitation of apoptotic particles in macrophages of described genotypes. Columns represent mean phagocytic index ± SEM. Control embryos (*n* = 6). *srp^3^* mutant embryos (*n* = 7). Asterisks indicate statistical significance versus control, as determined by Student’s *t*-test, ***p < 0.0001.

Given that macrophages of *srp* mutant appear abnormal and do not express SIMU, we tested their ability to phagocytose apoptotic cells. To evaluate their phagocytic capacity, we detected apoptotic particles with an anti-activated Dcp-1 antibody (*Drosophila* Caspase 3 homolog and a marker of apoptotic cells) (Figures 3A’,A’’,B’,B’’) and labeled macrophages with *srpGal4,UAScytGFP* (Figures 3A,A’’,B,B’’). We counted the number of apoptotic particles per macrophage, termed “phagocytic index” (explained in Materials and Methods). As expected, apoptotic particles were found inside GFP-positive macrophages in wild type embryos (Figures 3A’’,C). However, we could not detect any apoptotic particles inside macrophages of *srp* mutant (Figures 3B’’,C), suggesting their abnormal ability to phagocytose apoptotic cells.

**Figure 4:**
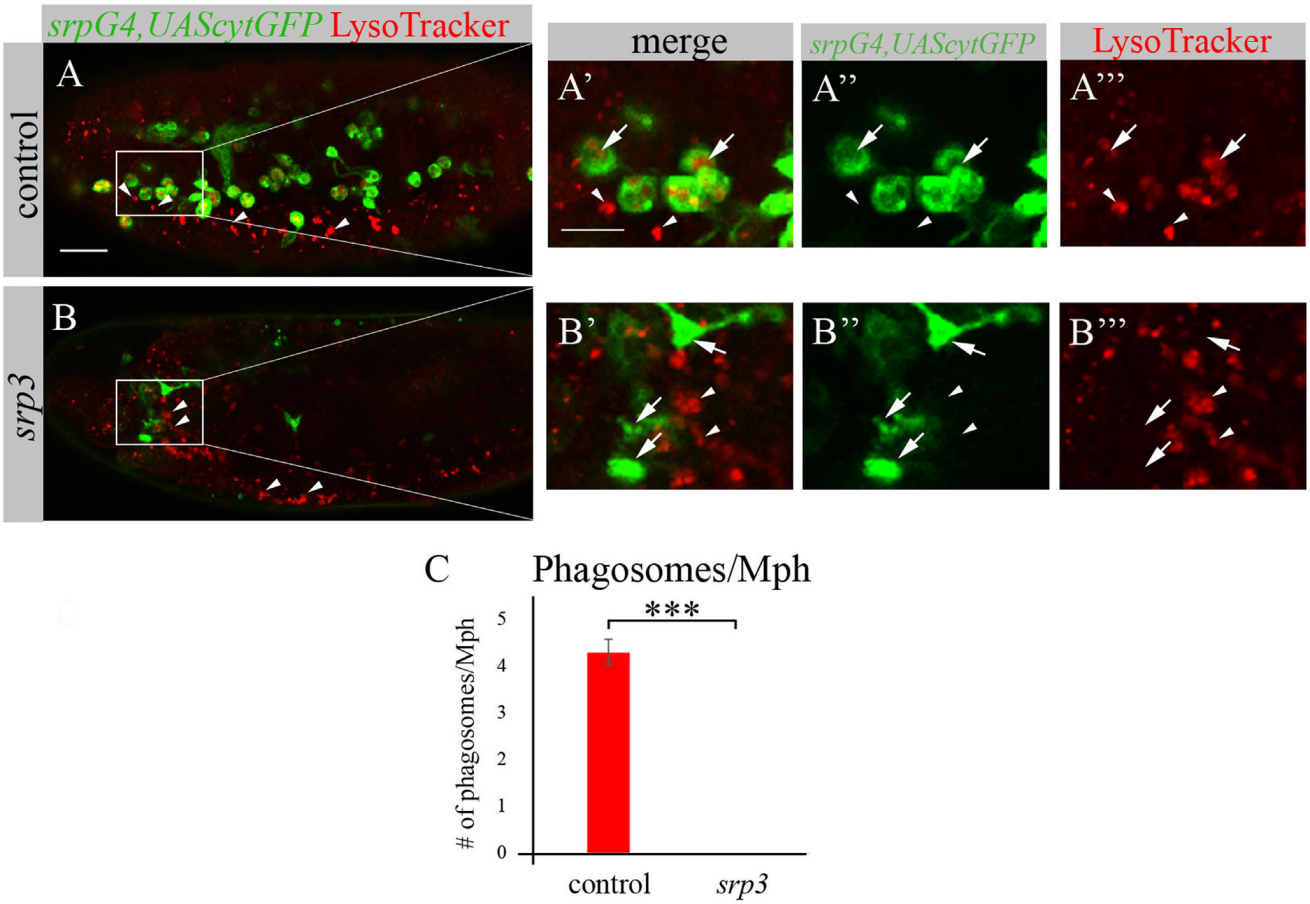
*srp* mutant macrophages are impaired in phagocytosis. **(A–B’’’)** Projections from confocal stacks of the stage 16 live embryos, lateral view. *srpGal4,UAScytGFP* marks macrophages in green. Phagolysosomes are labeled with LysoTracker (LT, red). **(A–A’’’)** Control *srpGal4,UAScytGFP* embryo shows numerous LT labeled phagolysosomes inside GFP-positive cells (arrows). **(B–B’’’)** In *srp^3^* mutant embryo there is no LT labeling in GFP-positive cells (arrows). LT labels glial phagolysosomes in the CNS on the ventral side (arrowheads). Bar, 20 µm. **(C)** Quantitation of LT-labeled phagolysosomes in macrophages of described genotypes. Columns represent mean number of phagosolysosomes per macrophage ± SEM. Control embryos (*n* = 5). *srp^3^* mutant embryos (*n* = 5). Asterisks indicate statistical significance versus control, as determined by Student’s *t*-test, ***p < 0.0001.

We took an additional approach to evaluate phagocytosis by macrophages using LysoTracker (LT), which specifically labels phagolysosomes/phagosomes (Figures 4A,A’,A’’’,B,B’,B’’’). Macrophages were labeled by *srpGal4,UAScytGFP* (Figures 4A–A’’,B–B’’) and contained multiple LT-labeled phagolysosomes in wild type embryos (Figures 4A,A’,C). However, in *srp* mutant embryos we could not detect any LT labeling in GFP-positive cells (Figures 4B,B’,C) once more demonstrating an impaired phagocytic ability of *srp* mutant macrophages.

### Srp Is Required for Expression of Drpr and Crq in Embryonic Macrophages

**Figure 5:**
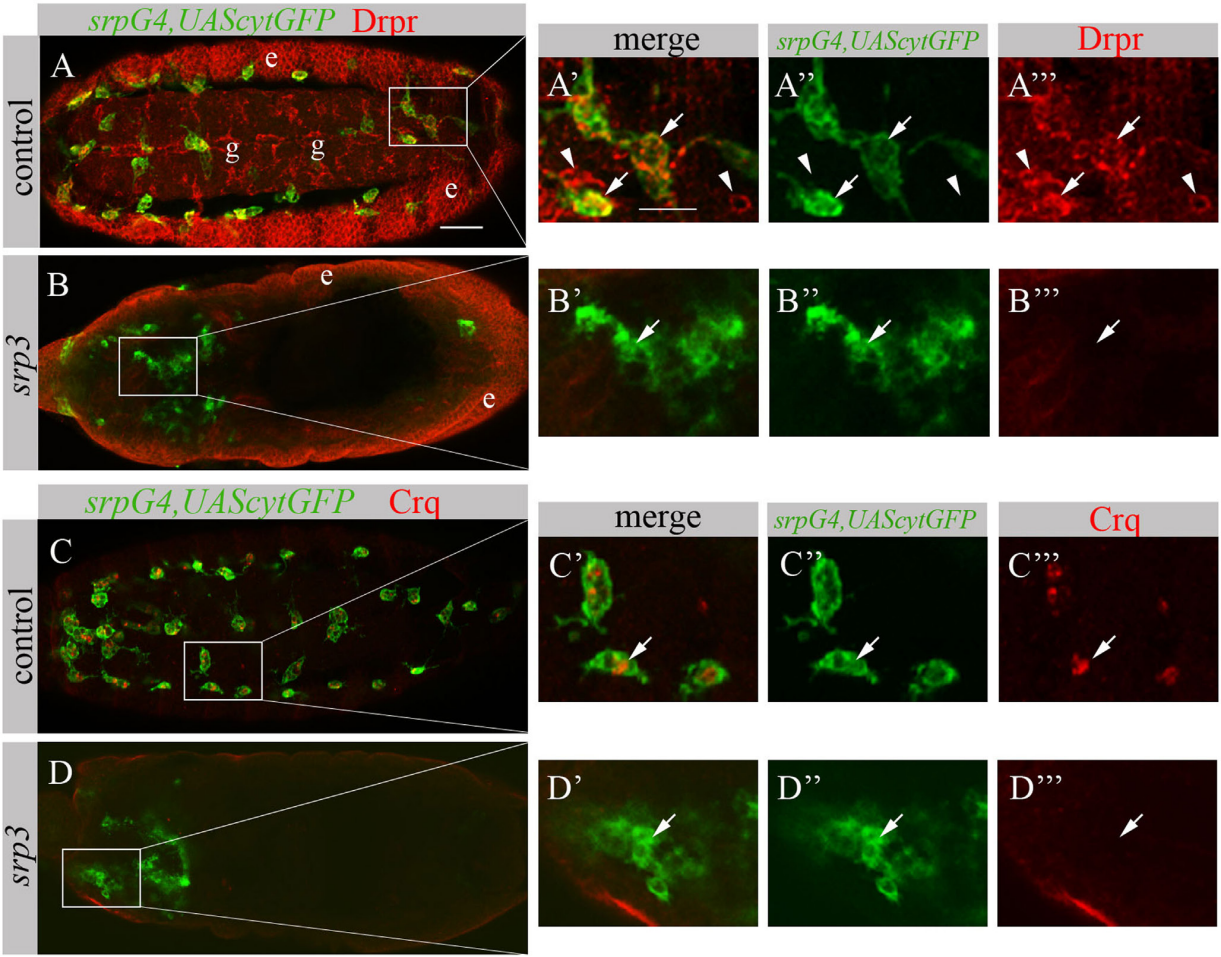
Srp is required for Drpr and Crq expression in embryonic macrophages. **(A–D’’’)** Projections from confocal stacks of the stage 16 embryos, ventral **(A–A’’’)** or dorsal view **(B–D’’’)**. Macrophages are labeled with *srpGal4,UAScytGFP* (green). **(A–B’’’)** Anti-Drpr in red, ectoderm (e) and glia (g). **(C–D’’’)** Anti-Crq in red. **(A’–A’’’, B’–B’’’, C’–C’’’, D’–D’’’)** Close up of rectangle areas in A,B,C and D respectively. **(A–A’’’)** Control *srpGal4,UAScytGFP* embryo expressing Drpr in GFP-positive macrophages (arrows) and GFP-negative glia (arrowheads). **(C–C’’’)** Control *srpGal4,UAScytGFP* embryo expressing Crq in GFP-positive macrophages (arrows). **(B–B’’’, D–D’’’)** *srp^3^* mutant embryos show no detectible Drpr **(B, B’’’)** and Crq **(D,D’’’)** staining in GFP-positive cells (arrows). Bar, 20 µm.

The impaired phagocytosis phenotype of *srp* mutant embryos appears much stronger than *simu* mutant phenotype (19), suggesting that additional phagocytic receptors may be affected by the absence of *srp*. To test this, we examined *srp* mutant embryos for the expression of two additional phagocytic receptors known to participate in apoptotic cell clearance by macrophages, Drpr and Crq (Figure 5). In control embryos, Drpr is specifically expressed in macrophages, glia and ectodermal cells as detected with anti-Drpr antibody (Figures 5A–A’’’). However, we were unable to detect any Drpr protein in macrophages of *srp* mutant labeled with *srpGal4,UAScytGFP*, though Drpr expression in the ectoderm remained normal (Figures 5B–B’’’). This reveals that Srp is required for Drpr expression in embryonic macrophages. Similarly, using an anti-Crq antibody (Figures 5C–D’’’) we found that Crq expression was undetectable in *srp* mutant embryos (Figures 5D–D’’’), indicating that Srp is required for Crq expression in embryonic macrophages as well.

### Srp Is Sufficient to Induce SIMU and Drpr Expression in Larval Glia

**Figure 6:**
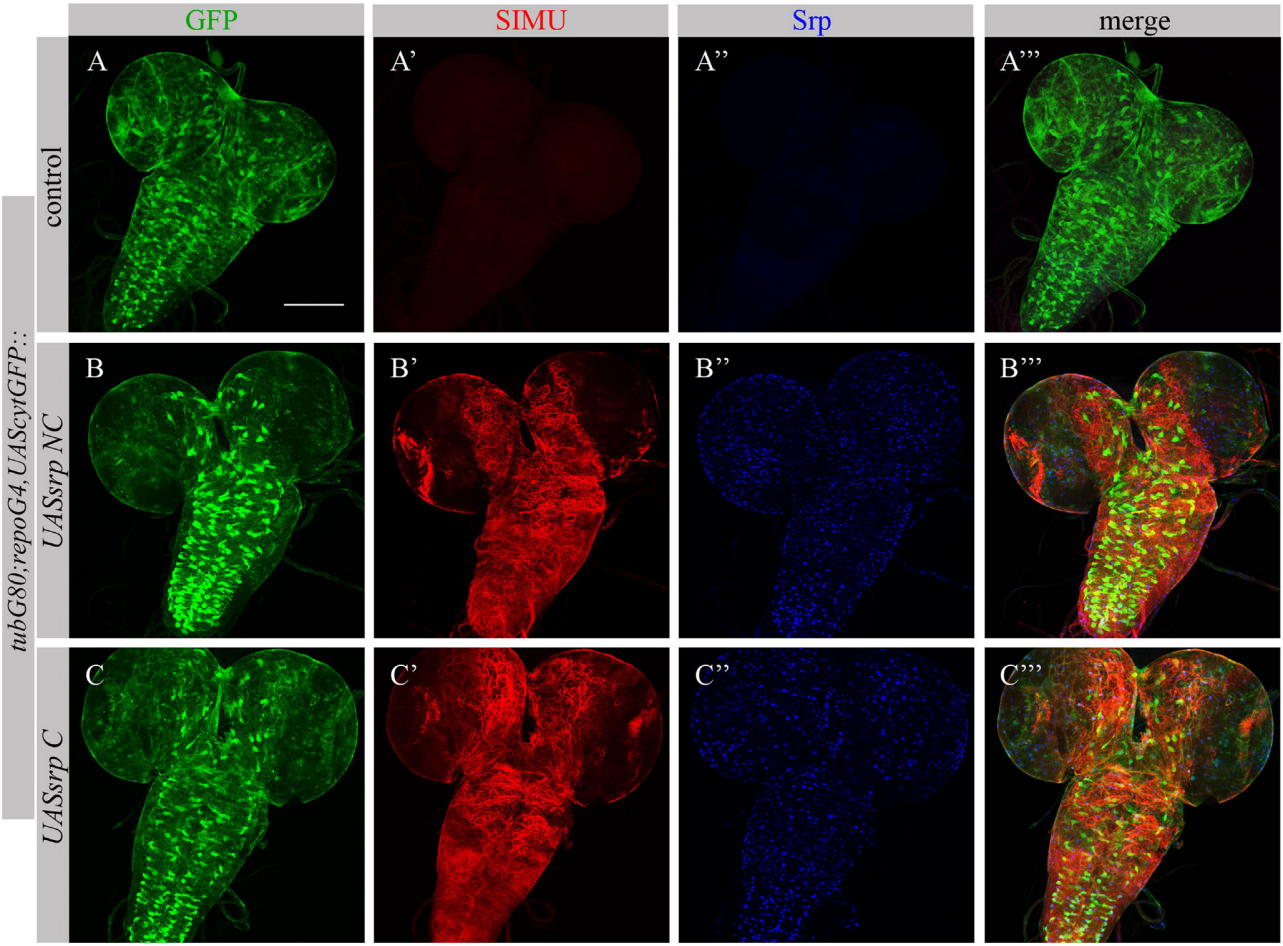
Each Srp isoform (SrpC and SrpNC) is sufficient to drive SIMU expression. **(A–C’’’)** Projections from confocal stacks of the 3^rd^ instar larval brains stained with anti-SIMU [red, **(A’,A’’’,B’,B’’’,C’,C’’’)**], glia are labeled with *repoGal4,UAScytGFP* [green, **(A,A’’’,B,B’’’,C,C’’’)**], anti-Srp [blue, **(A’’,A’’’,B’’,B’’’,C’’,C’’’)**]. Bar, 100 µm.

To test whether Srp is sufficient to induce SIMU expression, we ectopically expressed different isoforms of Srp, SrpNC (*UASsrpNC*) or SrpC (*UASsrpC*), in larval glial cells which normally do not express SIMU (Figure 6A’), using a *repoGal4* driver. *srp* ectopic expression in embryonic glia was prevented by a *tubGal80* temperature sensitive (ts) allele until the third instar larval stage. At this stage we moved the progeny (*repoGal4,UAScytGFP;tubGal80^ts^::UASsrpNC* or *repoGal4,UAScytGFP;tubGal80^ts^::UASsrpC*) from the permissive (18°C) to the restrictive (29°C) temperature of *tubGal80^ts^*. Dissected larval brains were stained with anti-Srp (Figures 6A”,A’’’,B’’,B’’’,C’’,C’’’) and anti-SIMU (Figures 6A’,A’’’,B’,B’’’,C’,C’’’) antibodies, which revealed that glial cells ectopically expressing Srp concomitantly expressed SIMU on their membranes (Figures 6B’’’,C’’’). These results demonstrate that *srp* is sufficient to drive SIMU expression. Both isoforms, SrpNC (Figures 6B–B’’’) and SrpC (Figures 6C–C’’’) were able to induce SIMU expression in larval glia (Figures 6B’,B’’’,C’,C’’’).

**Figure 7:**
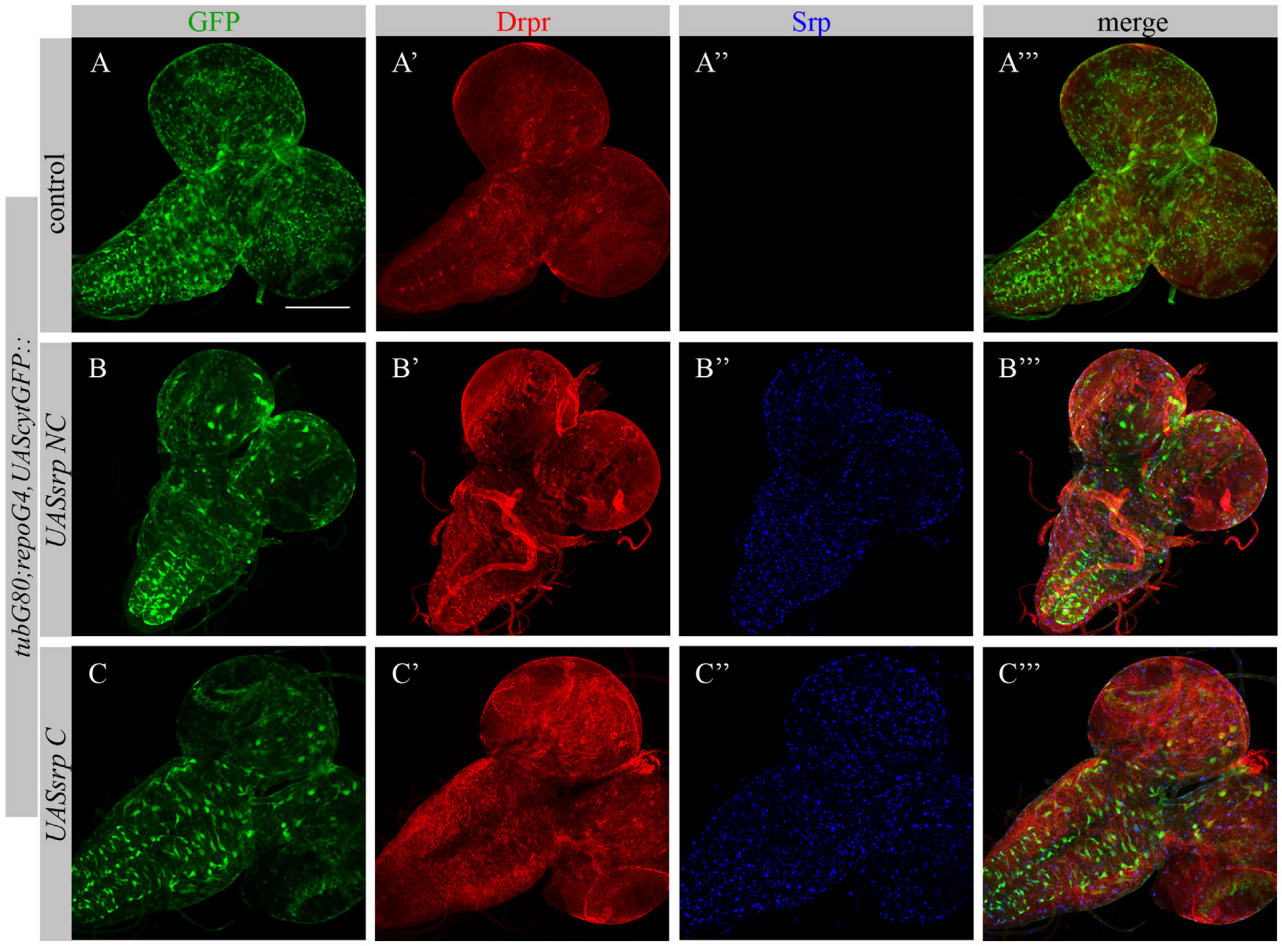
Each Srp isoform (SrpC and SrpNC) is sufficient to drive Drpr expression. **(A–C’’’)** Projections from confocal stacks of the 3^rd^ instar larval brains stained with anti-Drpr [red, **(A’,A’’’,B’,B’’’,C’,C’’’)**], glia are labeled with *repoGal4,UAScytGFP* [green, **(A,A’’’,B,B’’’,C,C’’’)**], anti-Srp [blue, **(A’’,A’’’,B’’,B’’’,C’’,C’’’)**]. Bar, 100 µm.

Following ectopic expression of Srp in larval glia, we tested appearance of Drpr in dissected larval brains (*repoGal4,UAScytGFP;tubGal80^ts^::UASsrpNC* or *repoGal4,UAScytGFP;tubGal80^ts^::UASsrpC*) by staining with anti-Srp and anti-Drpr antibodies. Compared to control glia (Figure 7A’), we detected more Drpr protein on membranes of glial cells ectopically expressing Srp (Figures 7B’,C’). Both isoforms SrpC and SrpNC were able to elevate Drpr expression in larval glia (Figures 7A’,A’’’,B’,B’’’,C’,C’’’), indicating that Srp is sufficient to induce Drpr expression. Importantly, it has been shown previously that SrpC is sufficient to induce Crq ectopic expression whereas SrpNC is not (28). These data suggest that the isoform C of Srp is sufficient to drive Drpr, Crq and SIMU expression, whereas the NC isoform can induce only SIMU and Drpr expression.

### GCM Is Dispensable for the Phagocytic Ability of Embryonic Macrophages

**Figure 8:**
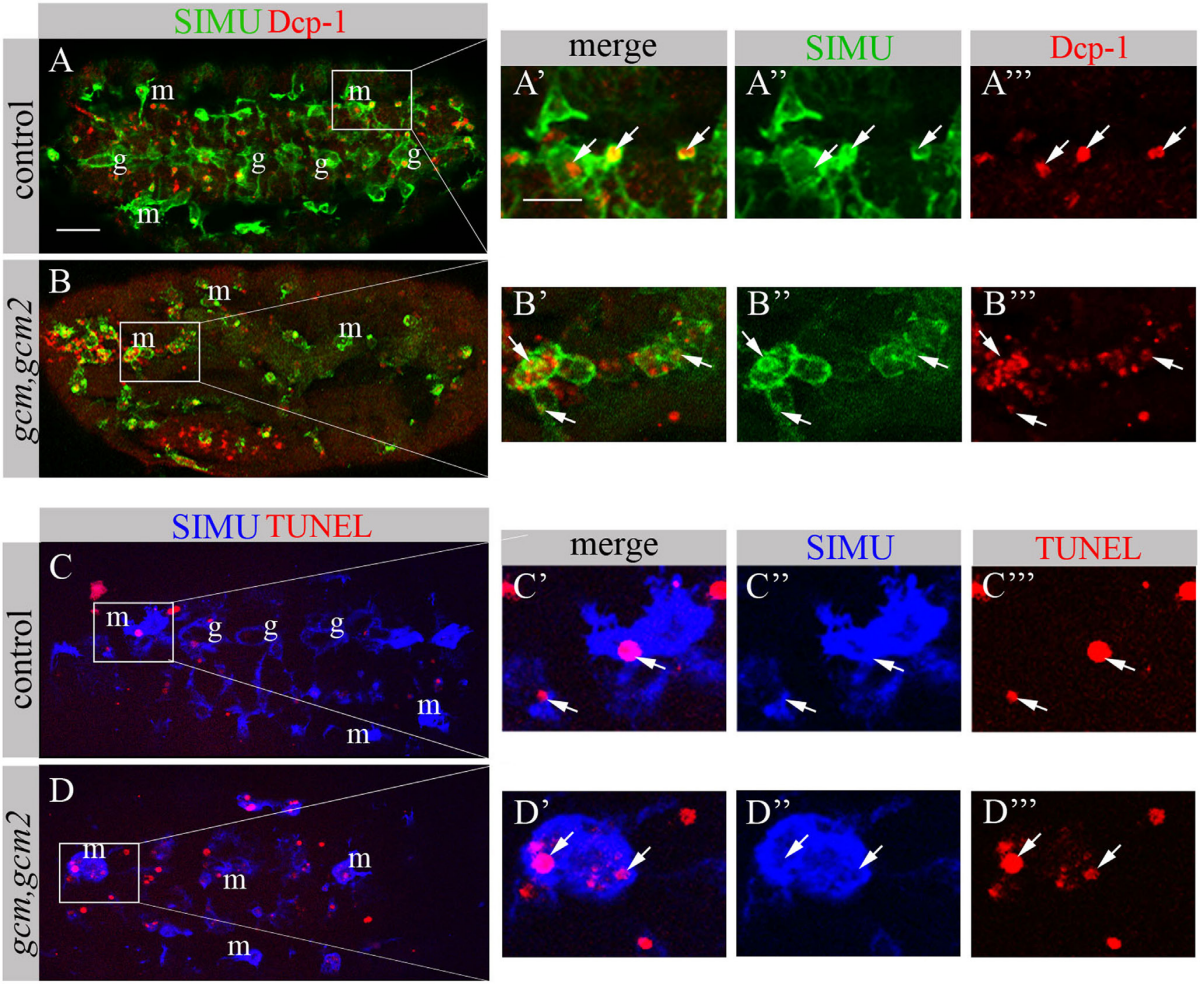
*gcm,gcm2* mutant macrophages phagocytose apoptotic cells. **(A–D’’’)** Projections from confocal stacks of the stage 16 embryo; **(A–B’’’)** apoptotic cells are in red (Dcp-1), SIMU protein with anti-SIMU in green. **(C–D’’’)** TUNEL in red, SIMU protein with anti-SIMU in blue. **(A–A’’’, C–C’’’)** Ventral view of control embryos displays SIMU expression in glia (g) and macrophages (m). Dcp-1 and TUNEL staining are found inside SIMU-labeled cells [**(A-A’’’, C–C’’’)** arrows]. **(B–B’’’, D–D’’’)** *gcm,gcm2* deficient embryos; no glia are labeled with SIMU but macrophages are (m). Dcp-1 and TUNEL staining are found inside SIMU-positive macrophages (arrows). Bar, 20 µm.

We have previously shown that GCM,GCM2 directly regulate *simu* expression only in embryonic glia but not in macrophages (26). Moreover, mutant *gcm,gcm2* macrophages still express SIMU, Drpr and Crq (26) (Figure 8). However, mutant embryos lacking *gcm* and *gcm2* contain a significantly lower number of embryonic macrophages (29, 34) suggesting that GCM,GCM2 are required for their proliferation, differentiation and/or survival. Nevertheless, whether GCM,GCM2 are essential for phagocytosis of apoptotic cells by macrophages has not been previously established. Using simultaneous labeling of embryonic macrophages with anti-SIMU and apoptotic cells with anti-Dcp-1 antibodies (Figures 8A–B’’’), we observed that *gcm,gcm2* mutant macrophages contain apoptotic particles inside them (Figures 8B–B’’’), demonstrating that they are capable of engulfing apoptotic cells. In addition, we performed terminal deoxynucleotidyl transferase dUTP nick and labeling (TUNEL) staining to label DNA fragments, characteristic of apoptotic cells in wild type (Figures 8C–C’’’) and *gcm,gcm2* mutant (Figures 8D–D’’’) embryos. Similarly to control embryos, in *gcm,gcm2* mutants SIMU-labeled macrophages contain TUNEL-positive particles confirming their ability to phagocytose apoptotic cells (Figures 8C–D’’’). These data demonstrate that GCM,GCM2 are not required for the phagocytic ability of embryonic macrophages.

**Figure 9:**
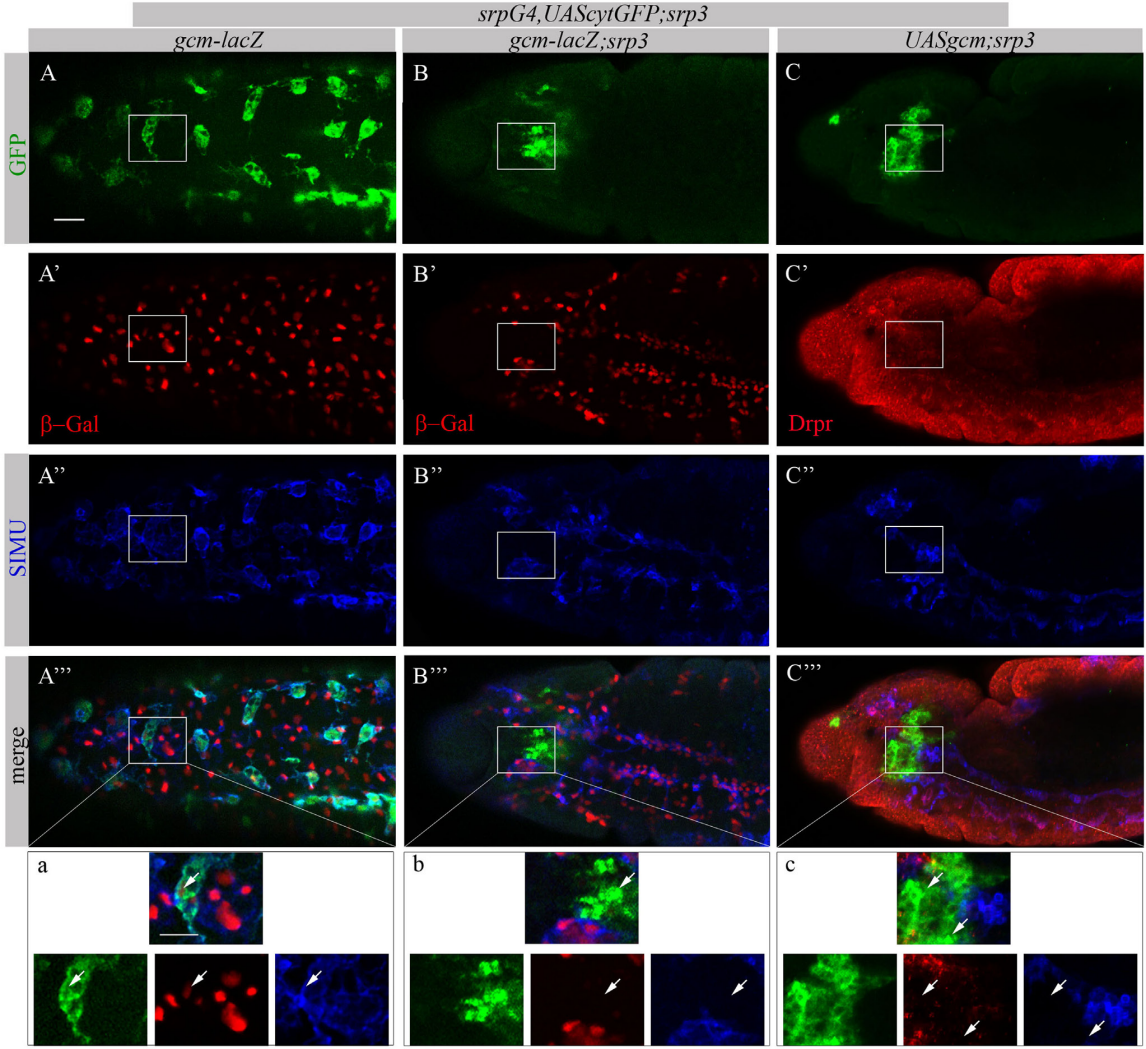
GCM is not sufficient to drive SIMU and Drpr expression in embryonic macrophages. **(A–c)** Projections from confocal stacks of the stage 16 embryos, **(A–a)** ventral view and **(B–c)** lateral view. Macrophages are labeled with *srpGal4,UAScytGFP* [green, **(A,A’’’,a,B,B’’’,b,C,C’’’,c)**] and SIMU protein with anti-SIMU [blue, **(A’’,A’’’,a,B’’,B’’’,b,C’’,C’’’,c)**]. **(A–b)** *gcm-lacZ* reporter in red and **(C–c)** Drpr with anti-Drpr in red. **(A–a)** Control *srpGal4,UAScytGFP* embryo. β-Gal and SIMU are expressed in GFP-positive macrophages (arrows). **(B–b)** *srp^3^* mutant embryo. No β-Gal and SIMU are detected in GFP-positive cells (arrows). **(C–c)** *srp^3^* mutant carrying GCM *(srpGal4::UASgcm)* in macrophages. No SIMU and Drpr are detected in GFP-positive cells (arrows). Bar, 20 µm.

Based on our previous study showing that GCM,GCM2 directly regulate *simu* expression in embryonic glia (26) we assumed that GCM,GCM2 may also induce *simu* expression in macrophages. However, since Srp binding sites in *simu* promoter are located in close proximity to the GCM binding site (L. Waltzer—personal communication) we hypothesized that it may sterically prevent GCM,GCM2 binding. To test this we aimed to examine whether GCM,GCM2 are able to induce SIMU expression in the absence of Srp (*srp* mutant). Normally GCM expression is not detected in *srp* mutants (Figures 9B’’’,b). Therefore, we expressed GCM (*UASgcm*) in *srp* mutant macrophages using the *srpGal4* driver (*srpGal4,UAScytGFP;srp^3^::UASgcm;srp^3^*) and tested whether it induces SIMU expression (Figure 9). No evident appearance of SIMU has been detected in *srp* mutant macrophages expressing GCM (Figures 9C’’’,c), indicating that GCM is not sufficient to induce *simu* in the absence of Srp. Moreover, in these embryos no Drpr expression was noticed in macrophages as well (Figures 9C’’’,c) demonstrating that GCM is also not sufficient to induce Drpr expression in embryonic macrophages.

### Each Phagocytic Receptor (SIMU, Drpr or Crq) Partially Rescues Distribution of *srp* Mutant Macrophages and Their Defects in Phagocytosis

**Figure 10:**
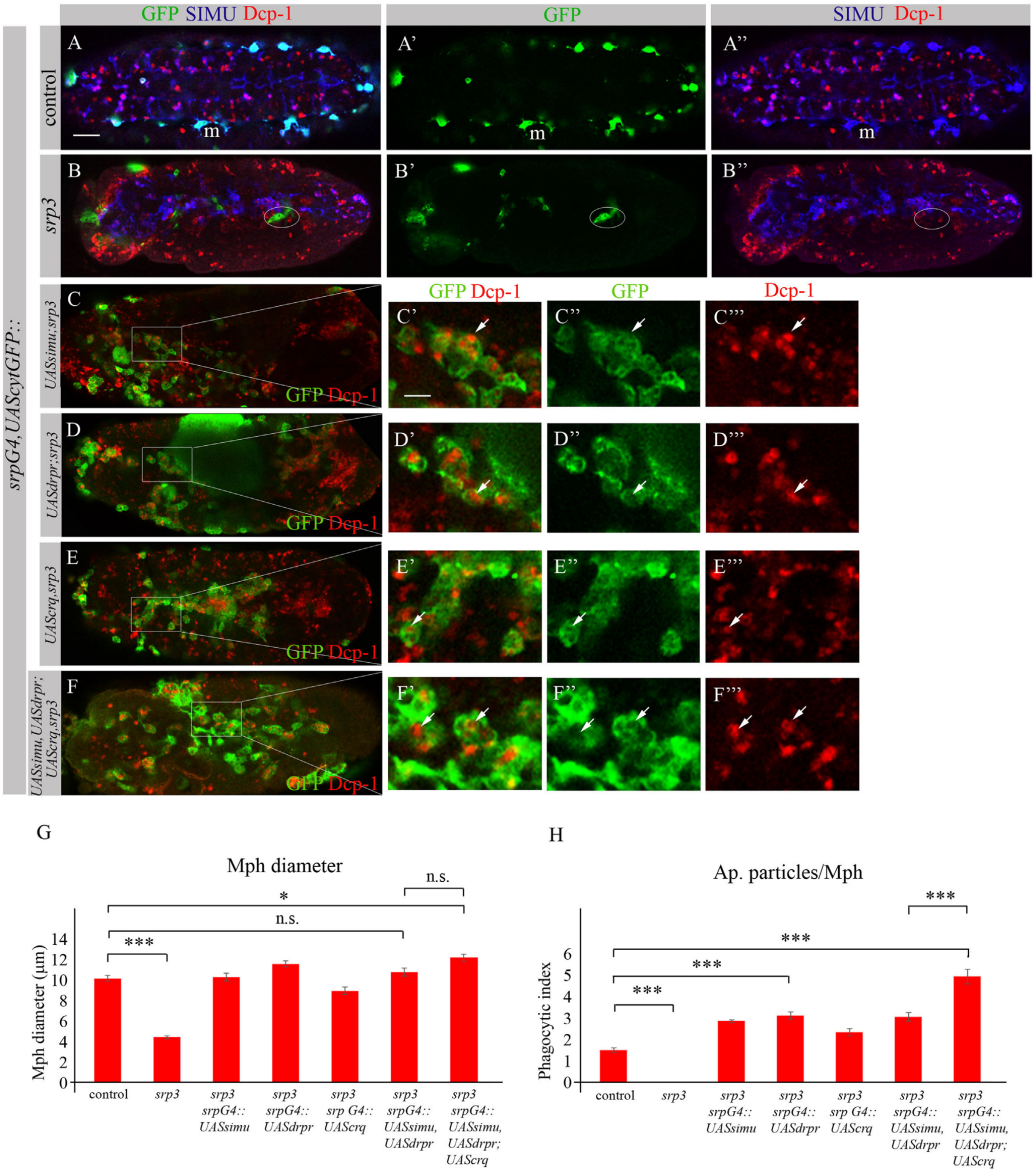
Each phagocytic receptor SIMU, Drpr or Crq rescues phagocytosis defects and distribution of *srp* mutant macrophages. **(A–F’’’)** Projections from confocal stacks of the stage 16 embryos. Macrophages are labeled with *srpGal4,UAScytGFP* [green, **(A,A’,B,B’,C–C’’,D–D’’,E–E’’,F–F’’)**], SIMU protein with anti-SIMU [blue, **(A,A’’,B,B’’)**] and apoptotic cells with anti-Dcp-1 [red, **(A,A’’,B,B’’,C,C’,C’’’,D,D’,D’’’,E,E’,E’’’,F,F’,F’’’)**]. **(A–A’’)** Control *srpGal4,UAScytGFP* embryo. **(B–B’’)** *srp^3^* mutant embryo. **(C–C’’’)** *srpGal4,UAScytGFP; srp^3^::UASsimu;srp^3^*. **(D–D’’’)** *srpGal4,UAScytGFP; srp^3^::UASdrpr;srp^3^*. **(E–E’’’)** *srpGal4,UAScytGFP; srp^3^::UAScrq,srp^3^*. **(F–F’’’)** *srpGal4,UAScytGFP; srp^3^::UASsimu,UASdrpr;UAScrq,srp^3^*. Bar, 20 µm. **(G)** Columns represent mean diameter of 10 macrophages in each embryo ± SEM of following genotypes: control embryos (*n* = 5), *srp^3^* mutant embryos (*n* = 7), *srpGal4,UAScytGFP; srp^3^::UASsimu;srp^3^* (*n* = 6), *srpGal4,UAScytGFP; srp^3^::UASdrpr;srp^3^* (*n* = 5), *srpGal4,UAScytGFP; srp^3^::UAScrq,srp^3^* (*n* = 8), *srpGal4,UAScytGFP; srp^3^::UASsimu,UASdrpr;srp^3^* (*n* = 8), *srpGal4,UAScytGFP; srp^3^::UASsimu,UASdrpr;UAScrq,srp^3^* (*n* = 6). Asterisks indicate statistical significance versus control, as determined by one-way ANOVA followed by Bonferroni *post hoc* test, ***p < 0.0001, *p < 0.05, n.s. >0.05. **(H)** Columns represent mean phagocytic index ± SEM of following genotypes: control embryos (*n* = 6), *srp^3^* mutant embryos (*n* = 7), *srpGal4,UAScytGFP; srp^3^::UASsimu;srp^3^* (*n* = 6), *srpGal4,UAScytGFP; srp^3^::UASdrpr;srp^3^* (*n* = 6), *srpGal4,UAScytGFP; srp^3^::UAScrq,srp^3^* (*n* = 8), *srpGal4,UAScytGFP; srp^3^::UASsimu,UASdrpr;srp^3^* (*n* = 8), *srpGal4,UAScytGFP; srp^3^::UASsimu,UASdrpr;UAScrq,srp^3^* (*n* = 6). Asterisks indicate statistical significance versus control, as determined by one-way ANOVA followed by Bonferroni *post hoc* test, ***p < 0.0001.

To investigate whether the impaired phagocytic ability of *srp* mutant macrophages results merely from the absence of the receptor expression, we performed rescue experiments. We expressed either SIMU (Figures 10C–C’’’), Drpr (Figures 10D–D’’’) or Crq (Figures 10E–E’’’) specifically in *srp* mutant macrophages using the *srpGal4* driver and tested their ability to phagocytose apoptotic cells by immunostaining with the anti-Dcp-1 antibody (Figure 10). Surprisingly, we found that *srp* mutant macrophages expressing SIMU, Drpr or Crq (*srpGal4,UAScytGFP;srp^3^::UASsimu;srp^3^* or *srpGal4,UAScytGFP;srp^3^::UASdrpr;srp^3^* or *srpGal4,UAScytGFP;srp^3^::UAScrq;srp^3^*) did not appear in clusters in the anterior part of the embryo like in *srp* mutants (Figures 9B–10B–B”) but were distributed throughout the embryo (Figures 10C–E). Moreover, their diameter was significantly bigger as compared to *srp* mutant macrophages (Figure 10G) and we found engulfed apoptotic cells inside these macrophages (Figures 10C’,D’,E’), indicating that they are capable of apoptotic cell clearance. We counted the number of apoptotic cells per macrophage (phagocytic index) in control, *srp* mutant and embryos carrying different rescue constructs (Figures 10A–E,H). These data revealed a significantly higher phagocytic index in *srp* mutant macrophages that express each receptor alone (Figure 10H), demonstrating that each phagocytic receptor, SIMU, Drpr or Crq is able by itself to partially rescue *srp* mutant phagocytosis phenotype. However, interestingly, in these rescued embryos significantly more apoptotic cells were detected inside macrophages compared to control embryos, demonstrating apoptotic cell accumulation. Importantly, we tested the effect of overexpression of each receptor in wild type macrophages using *srpGal4* driver (*srpGal4::UASsimu* or *srpGal4::UASdrpr* or *srpGal4::UAScrq*). Compared to control no significant difference was detected in phagocytic index of macrophages overexpressing each receptor (Figure S2 in Supplementary Material), suggesting that in wild type embryo phagocytic ability of macrophages is not affected by overexpression of phagocytic receptors and might be limited by the overall amount of apoptotic cells in the embryo.

The situation is different in *srp* mutant where compared to wild type much more apoptotic particles are present in the embryo (Figures 10A,B). When we tested co-expression of *simu* and *drpr* simultaneously in *srp* mutant macrophages using the *srpGal4* driver (*srpGal4,UAScytGFP;srp^3^::UASsimu,UASdrpr;srp^3^*) we obtained a similar amount of cells inside the macrophages as with each receptor alone (Figure 10H), suggesting the same engulfment/degradation ratio in clearance of apoptotic cells. However, when all three receptors SIMU, Drpr and Crq were expressed in *srp* mutant macrophages (*srpGal4,UAScytGFP;srp^3^::UASsimu,UASdrpr;UAScrq,srp^3^*), we observed a significantly higher phagocytic index as compared to each receptor alone (Figures 10F–F’’’,H), which indicates additional accumulation of apoptotic cells inside macrophages. This result may designate a higher engulfment/degradation ratio in macrophages expressing all three phagocytic receptors.

**Figure 11:**
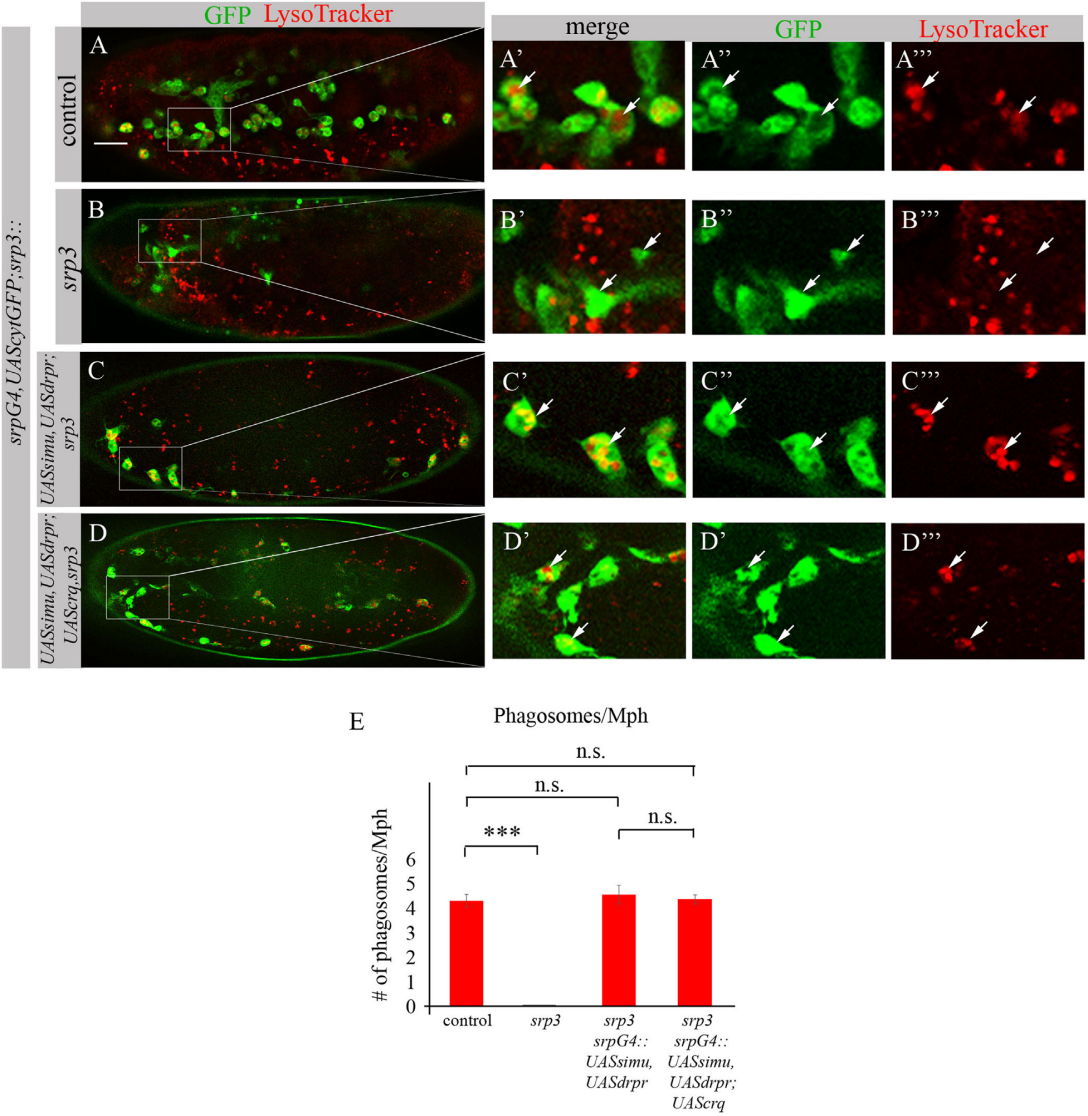
Phagocytic receptors SIMU, Drpr and/or Crq rescue phagocytosis defects of *srp* mutant macrophages. **(A–D’’’)** Stage 16 embryos. Macrophages are labeled with *srpGal4,UAScytGFP* [green, **(A–A’’,B–B’’,C–C’’,D–D’’)**], Phagolysosomes are labeled with LysoTracker (LT, red). **(A–A’’’)** Control *srpGal4,UAScytGFP* embryo. **(B–B’’’)** *srp^3^* mutant embryo. **(C–C’’’)** *srpGal4,UAScytGFP; srp^3^::UASsimu,UASdrpr;srp^3^*. **(D–D’’’)** *srpGal4,UAScytGFP; srp^3^::UASsimu,UASdrpr;UAScrq,srp^3^*. Bar, 20 µm. **(E)** Quantitation of LT-labeled phagolysosomes per macrophage of described genotypes. Columns represent mean number of phagosolysosomes ± SEM of following genotypes: control embryos (*n* = 5), *srp^3^* mutant embryos (*n* = 5), *srpGal4,UAScytGFP; srp^3^::UASsimu,UASdrpr;srp^3^* (*n* = 5), *srpGal4,UAScytGFP; srp^3^::UASsimu,UASdrpr;UAScrq,srp^3^* (*n* = 5). Asterisks indicate statistical significance versus control, as determined by one-way ANOVA followed by Bonferroni *post hoc* test, ***p < 0.0001, n.s. >0.05.

To test this assumption we evaluated degradation ability of *srp* mutant macrophages expressing SIMU and Drpr only or all three receptors SIMU, Drpr and Crq by quantifying LT-positive phagolysosomes in macrophages labeled with *srpGal4,cytGFP* (Figures 11A–E). No significant difference in the number of LT-positive phagosolysosomes was found between control macrophages (Figures 11A–A’’’,E) and *srp* mutant macrophages expressing two receptors (*srpGal4,UAScytGFP;srp^3^::UASsimu,UASdrpr;srp^3^*) (Figures 11C–C’’’,E) or three receptors together (*srpGal4,UAScytGFP;srp^3^::UASsimu,UASdrpr;UAScrq,srp^3^*) (Figures 11D–D’’’,E) indicating the similar degradation rate. These data strongly support our suggestion that the higher phagocytic index and bigger diameter of *srp* mutant macrophages expressing all three receptors than in *srp* mutant macrophages expressing only SIMU and Drpr is a result of the higher engulfment/degradation ratio and accumulation of apoptotic particles inside them.

## Discussion

Apoptotic cell clearance by “professional” and “non-professional” phagocytes plays a critical role during development of multicellular organisms. How the phagocytes acquire their ability to phagocytose apoptotic cells remains poorly understood. Key regulators of this process are phagocytic receptors for apoptotic cells that are specifically expressed on plasma membranes of phagocytes. However, the molecular mechanisms controlling expression of phagocytic receptors and therefore creating phagocytic ability of embryonic macrophages were unknown.

Using *Drosophila* embryonic macrophages as a model for development of “professional” phagocytes, we discovered that the GATA factor Srp is necessary for the specific expression of the phagocytic receptors SIMU, Drpr and Crq in these cells and sufficient to induce their expression in ectopic places. Therefore, the absence of Srp results in formation of abnormal macrophages lacking phagocytic receptors and thus incapable of apoptotic cell clearance. The defects in clearance can be substantially rescued by specific expression of each of the phagocytic receptors alone in embryonic macrophages. Surprisingly, we found that the presence of phagocytic receptors in *srp* mutant macrophages could also partially rescue their abnormal distribution. Interestingly, expression of each receptor, SIMU, Drpr or Crq resulted in comparable rescue of phagocytosis defects evaluated by phagocytic index. Similar phagocytic capacity of *srp* mutant macrophages expressing only one receptor suggests that each receptor is capable of persuading engulfment of apoptotic cells by macrophages. However, strikingly less apoptotic cells per macrophage are detected in the wild type embryos even if they overexpress the phagocytic receptors SIMU or Drpr or Crq. This could be explained by, in general, higher number of apoptotic cells present in *srp* mutant embryos and/or by their slower or impaired degradation inside phagolysosomes. Our results from the experiments with LT labeling of phagosomes suggest that higher number of engulfed apoptotic cells in the rescued macrophages is not accompanied by higher number of LT-positive phagolysosomes and therefore indicates slower degradation of engulfed apoptotic particles. This suggests that Srp may regulate expression of factors involved in the phagosome maturation process and therefore the degradation step in apoptotic cell clearance might be affected by its absence.

Furthermore, since SIMU and Crq are tethering receptors that are required for recognition and engulfment of apoptotic cells, expression of each receptor in *srp* mutant macrophages leads to the similar phenotype of engulfment and accumulation of apoptotic cells inside macrophages. However, we have previously shown that Drpr is mostly involved in degradation of apoptotic cells when SIMU and Crq are present (19). Our current results suggest that Drpr is capable of both engulfment and degradation of apoptotic particles when other receptors are missing, which is revealed by comparable phagocytic index in *srp* mutant macrophages that express Drpr alone with those that express SIMU or Crq. However, surprisingly, SIMU and Drpr joint expression demonstrates no additive effect on the phagocytic index. The possible explanation for this finding is that while SIMU allows more efficient engulfment compared to Drpr alone, Drpr itself permits faster degradation of the engulfed material. This is finally resulting in the similar phagocytic index of SIMU and Drpr joint expression to the expression of each one of them by itself. Interestingly though, when all three receptors are expressed (SIMU, Drpr and Crq), the amount of apoptotic cells per macrophage is significantly increased compared to SIMU and Drpr joint expression. These data suggest increased engulfment (by two tethering receptors SIMU and Crq) but limited degradation, which is mediated only by Drpr. Further confirmation of this conclusion comes from the same number of LT-positive phagolysosomes in the rescued macrophages expressing two receptors (SIMU and Drpr) and expressing all three receptors (SIMU, Drpr and Crq) demonstrating the same degradation rate and accumulation of more apoptotic cells in the macrophages expressing all three receptors. Taken together we demonstrate here that Srp creates phagocytic ability of embryonic macrophages by inducing balanced expression of the tethering receptors SIMU and Crq and the signaling receptor Drpr.

Our previous results revealed that GCM was not required for SIMU, Drpr and Crq expression in embryonic macrophages (26). Here we expanded our analysis on GCM role in apoptotic cell clearance by macrophages and demonstrate that GCM,GCM2 are not required for their function in phagocytosis of apoptotic cells. Significantly lower number of macrophages has been previously reported in *gcm* or *gcm,gcm2* double mutants compared to wild type (29, 34). Our data exhibit that the remaining macrophages express SIMU, Drpr and Crq. This finding suggests two possible scenarios: (1) the lack of *gcm,gcm2* may lead to apoptosis of macrophages resulting in the reduction of their number; Increased volume of apoptotic particles detected in *gcm,gcm2* mutants may also outcome from increased apoptosis of macrophages in addition to the abnormal apoptotic cell clearance by glial cells (26).

**Figure 12:**
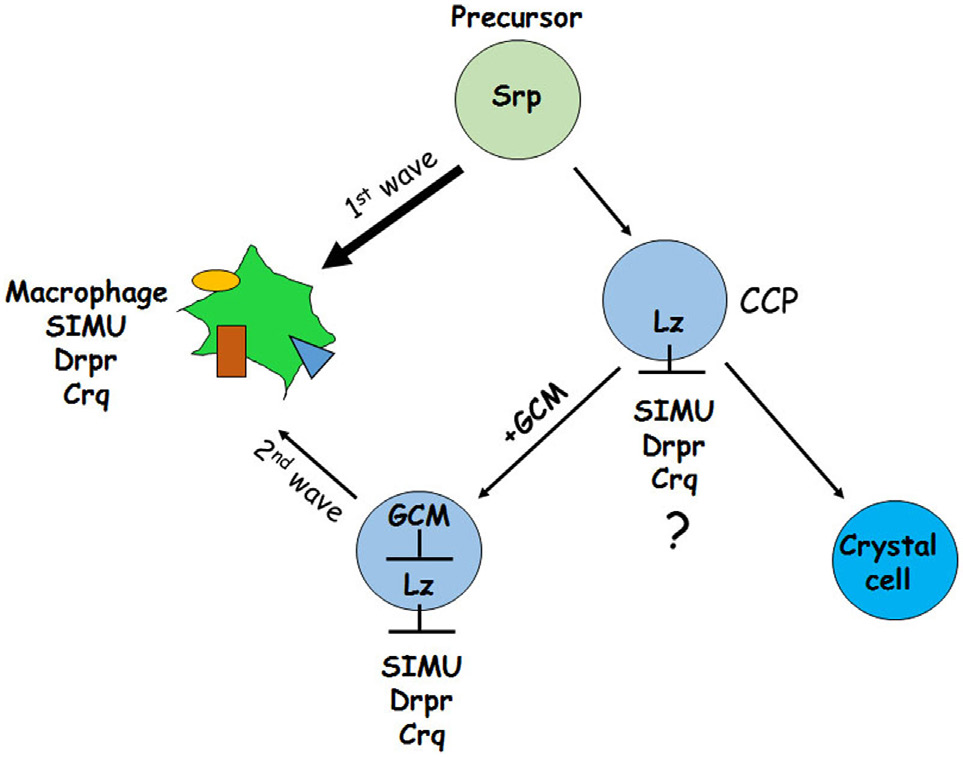
Schematic representation of two waves regulating development of embryonic macrophages. First wave starts from general Srp-positive hemocyte precursors and second develops from Lz-positive crystal cell precursors (CCPs). During the first wave Srp regulates SIMU, Drpr and Crq expression in plasmatocytes with no involvement of GCM,GCM2 and Lz. In the CCPs Lz expression likely (?) inhibits SIMU, Drpr and Crq expression resulting in formation of crystal cells that do not express these receptors. The second wave evolves from CCPs where GCM,GCM2 repress Lz, which allows expression of SIMU, Drpr and Crq and formation of macrophages. Srp may regulate additional factors involved in macrophage differentiation.

Another possibility (2) could be as shown in Figure 12. It has been demonstrated previously that GCM,GCM2 repress Lozenge (Lz)—a fate determinant factor of crystal cell development (35, 36). Two waves of plasmatocyte development were proposed: first starts from general Srp-positive hemocyte precursors and second develops from Lz-positive crystal cell precursors (CCPs) (35, 36). We suggest that during the first wave Srp regulates SIMU, Drpr and Crq expression in plasmatocytes independently of GCM,GCM2 and Lz. However, later on Lz-positive CCPs differentiate to crystal cells that do not express SIMU, Drpr and Crq, which may result from Lz function in these cells (Figure 12). The second wave of plasmatocyte formation evolving from CCPs requires GCM/GCM2, which repress Lz expression in part of CCPs that become macrophages (36) and express all three phagocytic receptors (Figure 12). If *gcm,gcm2* are absent, the second wave does not occur resulting in the reduced number of macrophages that express SIMU, Drpr and Crq compared to wild type embryos. We suggest that both possibilities can lead to the reduced number of macrophages in the *gcm,gcm2* mutant embryos.

The question why GCM,GCM2 do not regulate SIMU expression in embryonic macrophages through their binding sites remains open. We suggest that a repressor of GCM activity may act at early stages of embryogenesis in hemocyte precursors. During later stages of embryogenesis GCM,GCM2 directly induces *simu* expression in glial cells (26). Intriguingly, the same transcription factors GCM,GCM2 behave differently in two phagocytic cell populations glia and macrophages. This finding demonstrates that the phagocytic competence of different cell populations is determined by specific expression of phagocytic receptors that is regulated by diverse developmental programs. Using the *Drosophila* embryo as a model, we were able to expose basic molecular mechanisms essential for establishment of embryonic macrophages as potent phagocytes during development.

## Author Contributions

Conceived and designed the experiments: ES, BS, KH-M, FL-A, and EK. Performed the experiments: ES, BS, KH-M, and FL-A. Analyzed the data: ES, BS, KH-M, FL-A, and EK. Wrote the paper: EK.

## Conflict of Interest Statement

The authors declare that the research was conducted in the absence of any commercial or financial relationships that could be construed as a potential conflict of interest.
