# Supplementary figures and images for: Drosophila GATA Factor Serpent Establishes Phagocytic Ability of Embryonic Macrophages
Source: Front Immunol. 2018 Mar 8;9:266. doi: 10.3389/fimmu.2018.00266 (PMC5852079; doi:10.3389/fimmu.2018.00266)

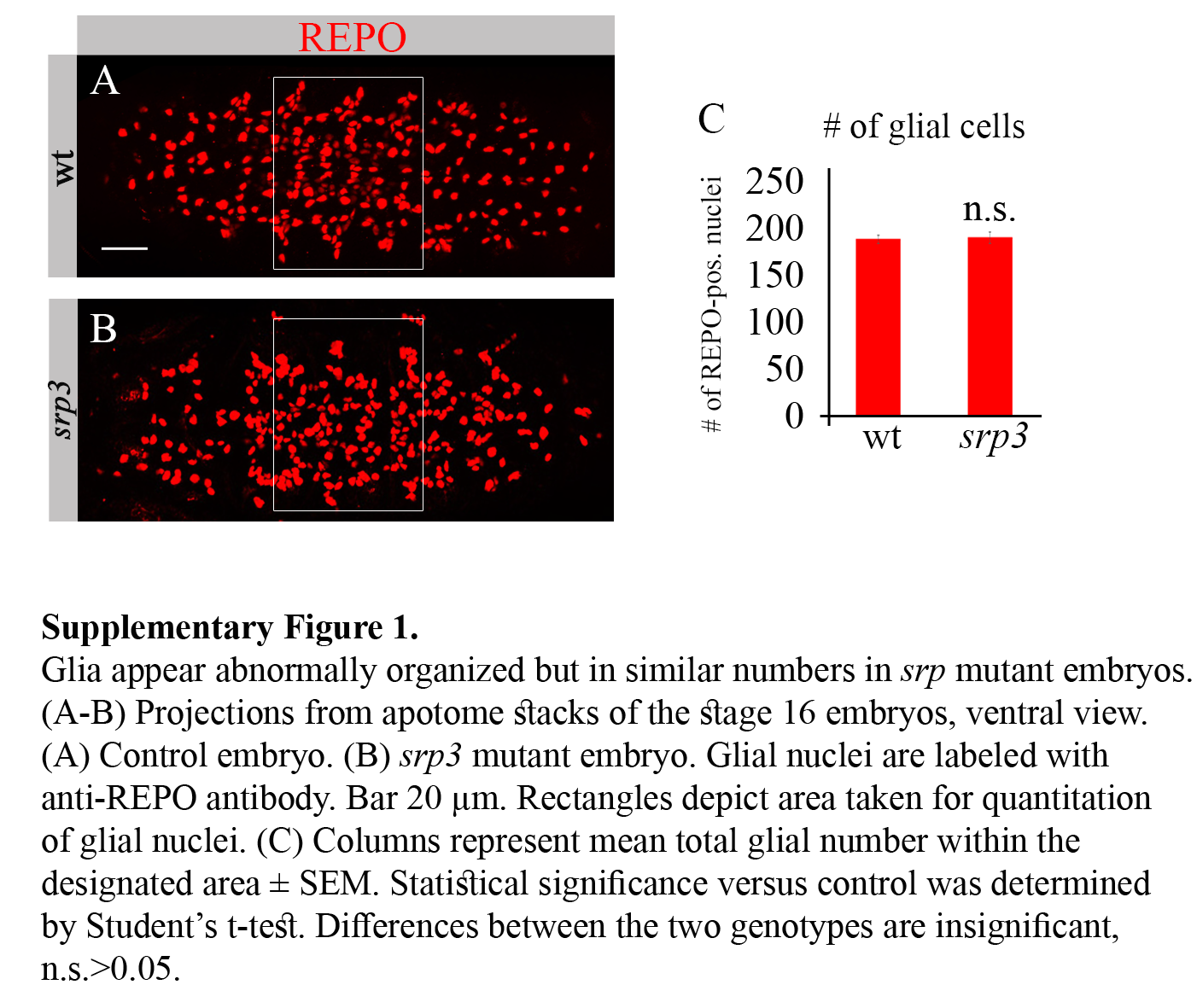

Supplement: Supplementary file 1 [file Image_1.tif]

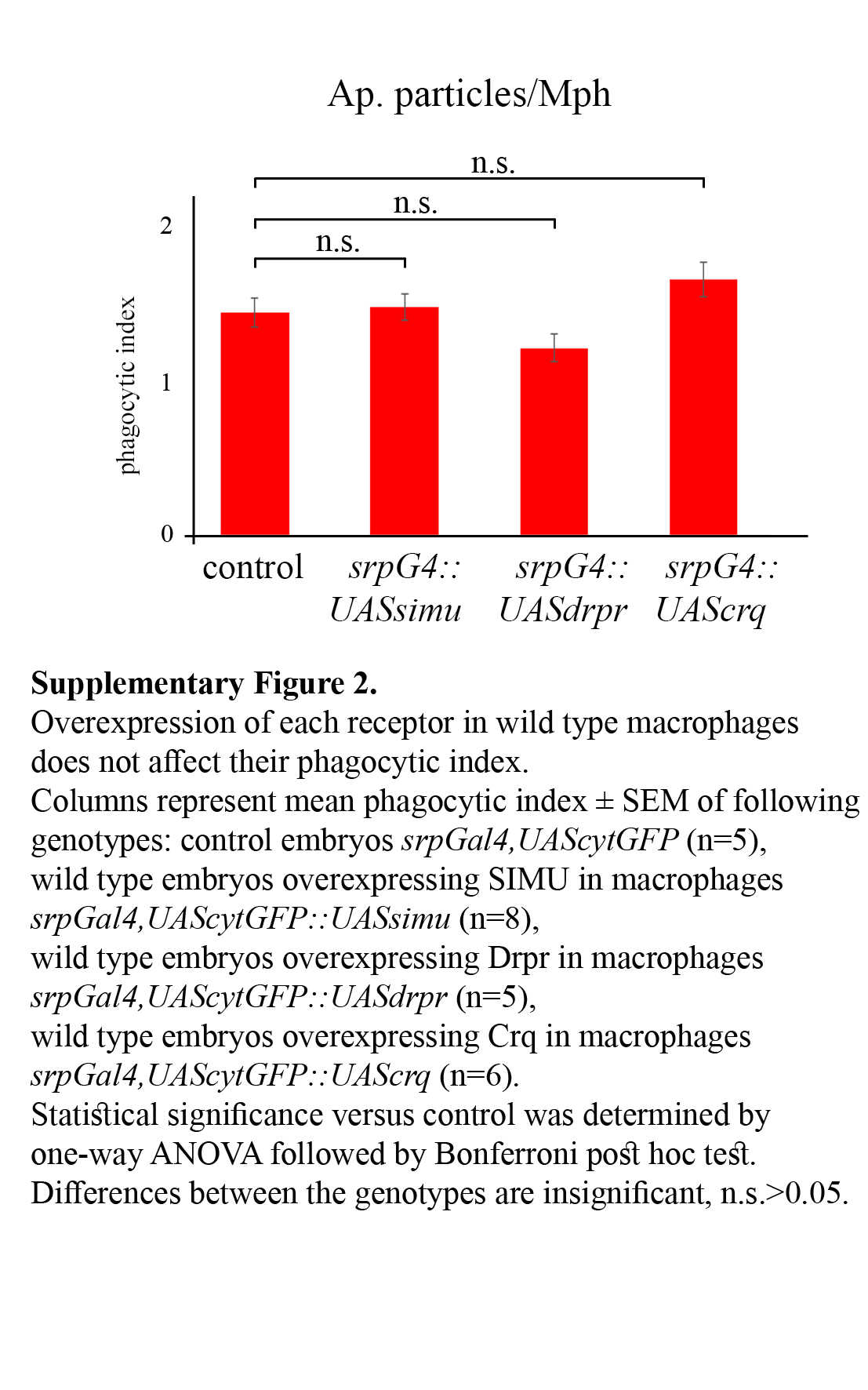

Supplement: Supplementary file 2 [file Image_2.tif]
